# Compositional and Functional Analysis of Golden and Brown Flaxseed: Nutrients, Bioactive Phytochemicals, Antioxidant Activity, and Cellular Responses

**DOI:** 10.3390/nu17213407

**Published:** 2025-10-29

**Authors:** Mariola Drozdowska, Ewelina Piasna-Słupecka, Klaudia Kmiecik, Ivo Doskocil, Barbora Lampova, Petr Smid, Barbara Domagała, Kinga Dziadek

**Affiliations:** 1 Department of Human Nutrition and Dietetics, University of Agriculture in Krakow, Balicka 122, 30-149 Krakow, Poland; mariola.drozdowska@urk.edu.pl (M.D.);; 2 Department of Microbiology, Nutrition and Dietetics, Faculty of Agrobiology, Food and Natural Resources, Czech University of Life Sciences Prague, Kamycka 129, Suchdol, 165 00 Prague, Czech Republiclampova@af.czu.cz (B.L.); 3 Department of Chemistry, Faculty of Agrobiology, Food and Natural Resources, Czech University of Life Sciences Prague, Kamycka 129, Suchdol, 165 00 Prague, Czech Republic; 4 Department of Horticulture, Faculty of Biotechnology and Horticulture, University of Agriculture in Krakow, 29 Listopada St. 54, 31-425 Krakow, Poland; barbara.domagala@urk.edu.pl

**Keywords:** golden, brown flaxseed, *Linum usitatissimum* L., nutrients, polyphenols, antioxidant activity, anticancer activity

## Abstract

Background: Flaxseed (*Linum usitatissimum* L.) represents a unique source of bioactive compounds with demonstrated health benefits. The main aim of the research was to investigate the chemical composition, content of bioactive compounds and biological activities of various types of flaxseed and their defatted forms. Methods: Proximate composition (crude fat, protein, ash, digestible carbohydrates, fiber) was determined, and fatty acid profiles were analyzed via GC-MS (gas chromatography–mass spectrometry). Mineral content was measured by atomic absorption spectrometry, while total and individual polyphenols were quantified spectrophotometrically and by HPLC (high-performance liquid chromatography). Antioxidant activity was assessed using three assays. In vitro functional assays evaluated the effects of flaxseed extracts on lactic acid bacteria adhesion in two cellular models, nitric oxide production in liposaccharide (LPS)-stimulated RAW 264.7 macrophages, proliferation and apoptosis of MCF-7 breast cancer cells. Results: Significant differences (*p* ≤ 0.05) were observed in the proximate composition: brown flaxseed exhibited the highest crude fat content, whereas defatted seeds had higher levels of digestible carbohydrates and ash. α-Linolenic acid was the dominant fatty acid, with the highest concentration in defatted golden flaxseed. Defatted forms generally displayed increased mineral concentrations, particularly calcium, magnesium, potassium, and iron. The polyphenolic content and antioxidant activity were highest in defatted brown flaxseed, which also exhibited the greatest diversity of individual polyphenols. Flaxseed extracts modulated the adhesion of lactic acid bacteria, reduced the production of nitric oxide in RAW 264.7 macrophages, inhibited the proliferation of MCF-7 breast cancer cells in a dose- and time-dependent manner, and induced apoptosis of the mentioned cells. Conclusions: Flaxseed, especially the brown type, could be a promising source of bioactive compounds with antioxidant, anti-inflammatory and anticancer potential, supporting its use in nutritional and functional applications.

## 1. Introduction

Common flax (*Linum usitatissumum* L.) is an annual plant belonging to the *Linaceae family*, which comprises more than 200 species. Flax is generally classified into two main varieties: brown and golden. Golden flax is adapted to extremely cold environments, whereas brown flax grows better under warmer and more humid conditions. The small, flat, oval seeds vary in color from golden yellow to reddish-brown [1]. Traditionally, flaxseed (linseed) has been utilized for textile production, varnishes, and medicinal application, including the treatment of respiratory, digestive, and dermatological disorders [2]. Flaxseed is regarded as a functional food due to its high concentration of bioactive compounds such as polyunsaturated fatty acids (omega-3 and omega-6), vitamins, minerals, peptides, phenolic acids, flavonoids, and lignans. These bioactives have been associated with hypolipidemic, anti-atherosclerotic, anti-cholesterolemic, anti-inflammatory, and anticancer effects, among others [3].

Flax is one of the richest plant sources of essential *n*-3 and *n*-6 fatty acids, primarily α-linolenic acid (ALA, C18:3 *n*-3) and linoleic acid (LA, C18:2 *n*-6) [4]. Physiologically, omega-3 fatty acids play a key role in supporting normal prenatal development, preventing preterm birth and perinatal death, and reducing the risk of metabolic syndrome, obesity, type 2 diabetes, and cardiovascular diseases such as ischemic heart disease, atherosclerosis, and myocardial infarction. Maintaining an appropriate *n*-6:*n*-3 fatty acid ratio ideally around 5:1, ensures anti-inflammatory, antioxidant, anti-allergic, and anticancer effects [5]. Eicosapentaenoic acid (EPA), derived from ALA, modulates inflammation through its role as a prostaglandin precursor involved in immune regulation and anti-inflammatory responses. Oral supplementation with flaxseed oil has been shown to alleviate acute pneumonia, reduce inflammatory markers, and exhibit antibacterial properties [2].

Another key characteristic of flaxseed is its strong antioxidant potential, attributable to its high levels of polyunsaturated fatty acid (PUFAs), tocopherols, polyphenols, and phytosterols. Flaxseed contains between 75 and 800 times higher concentration of lignans than most other seeds, predominantly in the form of secoisolariciresinol diglucoside (SDG), metabolized by gut microbiota into enterodiol (ED) and enterolactone (EL). The general health-promoting properties of enterolignans are due to their antioxidant activity and ability to modulate cellular pathways, including in cancer cells [6]. The soluble fiber in flaxseed, especially mucilage gum, helps nourish probiotics like *Lactobacillus* and *Bifidobacteria*, supporting a healthy gut microbiome. Flaxseed can contribute to improved digestion, better immune function and enhanced nutrient absorption by promoting the growth of beneficial gut microbiota.

Flaxseed has been evaluated in comparison with other oilseeds, particularly chia (*Salvia hispanica* L.), hemp (*Cannabis sativa* L.) and sesame (*Sesamum indicum* L.), which are also recognized as valuable sources of polyunsaturated fatty acids and bioactive compounds. Recent studies have indicated that chia seeds contain slightly higher levels of total dietary fiber and comparable levels of α-linolenic acid. In contrast, hemp seeds are distinguished by their high-quality protein and favorable amino acid profile, whereas sesame seeds are distinguished by their high lignan content, which is primarily composed of sesamin and sesamolin [7,8]. Despite these nutritional similarities, flaxseed remains the richest known dietary source of secoisolariciresinol diglucoside (SDG) and one of the most concentrated plant sources of ALA, which may explain its particularly strong antioxidant and anti-inflammatory potential compared with other oilseeds [2]. Therefore, situating flaxseed within the broader context of oil-bearing seeds provides a more comprehensive understanding of its unique health-promoting properties and supports its growing role in functional food applications [9]. Considering the current state of knowledge, it was scientifically justified to undertake studies designed to elucidate and compare the potential in vitro anticancer and anti-inflammatory activities of golden and brown flaxseed. Investigations assessing the in vitro adhesion capacity of probiotics to intestinal epithelial cells when exposed to flaxseed may provide further insight into its functional food potential and its role in supporting intestinal health. The antioxidant, anticancer and anti-inflammatory properties of plants are attributable to the synergistic action (enhancement or inhibition of mutual interactions) of several phytochemicals present in these foods. This combined bioactivity markedly increases the potential health benefits of flaxseed, underscoring the relevance of flaxseeds as a valuable addition to a balanced diet and highlighting the scientific and practical importance of the present research.

## 2. Materials and Methods

### 2.1. Plant Material

Seeds of two varieties of common flax (*Linum usitatissumum* L.) were used as experimental material: the golden flaxseed, defatted golden flaxseed, brown flaxseed, and defatted brown flaxseed. Flaxseeds were obtained from varieties growing in Poland and purchased on the local Polish market.

### 2.2. Proximate Composition

The chemical composition of the flaxseed was determined following AOAC procedures (Association of Official Analytical Collaboration International) [10]. Protein, fat, and ash contents were analyzed according to methods 950.36, 935.38, and 930.05, respectively. Dietary fiber was quantified using a Megazyme assay kit (Megazyme International, Wicklow, Ireland). The digestible carbohydrate content was calculated using the following equation: digestible carbohydrates = 100 − (protein + crude fat + ash + dietary fiber) [11].

### 2.3. Fatty Acid Profile

Fatty acid profiles were determined by gas chromatography–mass spectrometry (GC–MS) after lipid extraction from flaxseed samples. Free fatty acids were converted to fatty acid methyl esters (FAMEs) using boron trifluoride in methanol (BF_3_/MeOH) according to Morrison and Smith [12]. The FAMEs were analyzed using a GC–MS system equipped with a capillary column, following standard chromatographic conditions (See Supplementary Materials for details).

### 2.4. Minerals Content

Flaxseed samples were digested by microwave-assisted wet mineralization using 65% HNO_3_. The concentrations of metal ions (Ca^2+^, Mg^2+^, K^+^, Na^+^, Fe^3+^, Zn^2+^, Cu^2+^, Mn^2+^) were determined by flame Atomic Absorption Spectrometry (Varian AA240FS) with a Sample Introduction Pump System (SIPS-20). Measurements were carried out at element-specific wavelengths under optimized instrument conditions (see Supplementary Materials for details).

### 2.5. Extracts Preparation

Fresh flaxseed samples (1 g) were extracted with 80 mL of 70% to determine total polyphenol content and antioxidant activity. Plant material was extracted with 40 mL of 0.1% formic acid dissolved in 70% HPLC grade methanol (*w*/*v*) in order to identify the polyphenolic compounds by HPLC. Then, the samples were shaken at room temperature for 2 h (Elpin Plus, water bath shaker type 357, Lubawa, Poland) and centrifuged at 1500 rpm for 15 min (centrifuge type MPW-340, Warsaw, Poland). The supernatant was collected and stored at −20 °C until further analysis.

### 2.6. Determination of Selected Bioactive Compounds

Vitamin C content, expressed as the sum of ascorbic and dehydroascorbic acids, was determined by the Tillmans method in Pijanowski’s modification [11]. In this procedure, dehydroascorbic acid is reduced to ascorbic acid using sodium sulfide, and the total ascorbic acid is quantified by titration with 2,6-dichlorophenolindophenol. An acetone–hexane mixture (4:6 *v*/*v*) was used for extraction of carotenoids from the samples according to the PN-EN 12136:2000 [13]. An approximately amount of the sample (2 g) was mixed with 25 mL of the acetone–hexane mixture and thoroughly vortexed to extract the carotenoids. The mixture was then centrifuged, and the upper hexane layer containing the carotenoids was collected. The concentration of total carotenoids was measured spectrophotometrically at 450 nm (spectrophotometer UV-1800, Ray-Leigh, Beijing Beifen-Ruili Analytical Instrument Co., Ltd., Beijing, China). The content of total polyphenols in the extracts was estimated spectrophotometrically at 760 nm (spectrophotometer, as above) using the Folin–Ciocalteu reagent (Sigma Aldrich, St. Louis, MO, USA) [14]. The results were expressed as mg of chlorogenic acid equivalents per 100 g of sample (mg ChlA eq./100 g sample).

### 2.7. HPLC Analysis of Polyphenols

The profile of polyphenolic compounds was determined by liquid chromatography (HPLC), in pre-prepared methanolic extracts. Separation was performed on a C18 column (Phenomenex, Torrance, CA, USA) using a binary mobile phase consisting of 0.1% formic acid in water and methanol. Identification and quantification were carried out using external standards, and data were processed with Lab Solutions software (Shimadzu, Kyoto, Japan). Detailed analytical conditions and gradient parameters are provided in the Supplementary Materials.

### 2.8. Determination of Antioxidant Activity

The antioxidative activity of the samples was measured using three methods: ABTS^●^* (2,20-azinobis-(3-ethylbenzothiazoline-6-sulfonic acid)), DPPH (2,2-diphenyl-1-picrylhydrazyl) and FRAP (ferric reducing antioxidant power). The assays followed the methodology described by Dziadek et al. [15]. The ABTS^●^* and DPPH assays assessed the ability of sample antioxidants to scavenge free radicals, while the FRAP method measured the reducing power of antioxidants by converting Fe^3+^ to Fe^2+^. All assays utilized a UV-Vis spectrophotometer. Results were determined using a calibration curve and expressed as micromoles of Trolox equivalent per gram of sample (TEAC).

### 2.9. Cell Culture

The human epithelial cell line Caco-2 derived from a colon carcinoma (ATCC^®^—American Type Culture Collection, HTB-37™, Manassas, VA, USA), the human colorectal adenocarcinoma cell line HT-29 (ATCC^®^ HTB-38™, Manassas, VA, USA), HT-29-MTX cell line, a derivative of HT-29 that produces mucin (Sigma Aldrich 12040401, Saint Louis, MO, USA) and the human breast adenocarcinoma MCF-7 cell line (hormone-dependent, ATCC^®^ HTB-22^TM,^, Manassas, VA, USA) were cultured in Eagle’s Minimum Essential Medium (EMEM) supplemented with 10% fetal bovine serum (FBS) and antibiotics. Cultures were maintained under standard conditions (37 °C, 5% CO_2_, 95% humidity) in an incubator (NuAire, Plymouth, MN, USA). The murine macrophage cell line RAW 264.7 (ATCC TIB-71, Manassas, VA, USA) was cultured in RPMI-1640 medium (BioWest, France), containing 10% FBS, 1% nonessential amino acids (BioWest, France) and 5% glucose (Sigma-Aldrich, Saint Louis, MO, USA), under the same incubation conditions.

### 2.10. Extraction for Cell Culture Preparation

Homogenized samples (0.6 g) were extracted with 10 mL of 80% ethanol (ethanol:water, 80:20, *v*/*v*) in 50 mL screw-capped Falcon tubes. The mixtures were vortexed (Basic 3, IKA Werke, Staufen, Germany), sonicated for 15 min (PS 04, Powersonic-Notus Ltd., Vráble, Slovakia), and incubated overnight at 4 °C. Following centrifugation at 8228× *g* for 5 min (5810R, Eppendorf, Hamburg, Germany), an aliquot equivalent to 512 mg of sample was taken and adjusted to 10 mL with the extraction solvent.

### 2.11. Adhesion of Lactic Acid Bacteria

The effect of flaxseed ethanol extracts on adhesion was determined using human colorectal cell lines Caco-2, HT-29 and HT-29-MTX. A mixed cell culture of Caco-2 and HT-29 cells were seeded into 96-well plates at a density of 10^4^ cells/mL and cultured for 14 days. *Lactobacillus acidophilus* and *Lactobacillus reuteri* were prepared at a concentration of 10^7^ colony-forming units (CFU)/mL and stained with fluorescein isothiocyanate (25 μg/mL, Life Technologies, Carlsbad, CA, USA) for 30 min at 37 °C in a dark environment. The bacteria were then co-cultured with test samples at 100, 50, and 25 µg/mL concentrations for 120 min. Wells were washed three times with phosphate-buffered saline (PBS), and fluorescence was measured using a Tecan Spark plate reader (Tecan Group, Männedorf, Switzerland) at excitation 495 nm and emission 519 nm.

The percentage (%) of adhered bacteria was determined using the formula:

Adhesion ratio (%) = 100 × A/A0

A is the fluorescence intensity after adherence of *L. reuteri* or *L. acidophilus* to the cell culture; A0 is the initial fluorescence value measured after removing unbound bacteria.

### 2.12. In Vitro Anti-Inflammatory Activity

RAW 264.7 cells were seeded into 96-well plates at a density of 1 × 10^5^ cells/100 µL. After 2 h of incubation, extracts at concentrations of 256, 128, 64, 32, and 16 µg/mL and lipopolysaccharide (LPS, 1 µg/mL) were added and incubated for 24 h. Then, 50 µL of supernatant was mixed with Griess reagent (Sigma-Aldrich, Saint Louis, MO, USA), and absorbance was measured at 540 nm. Results were determined using a sodium nitrite calibration curve. After the nitric oxide (NO) inhibition test, the cytotoxicity test was performed according to Mosman [16]. Cells were incubated with MTT (1 µg/mL) for 2 h, and absorbance was measured at 495 nm.

### 2.13. Cell Proliferation

Cell proliferation was evaluated using the 5-bromo-2′-deoxyuridine BrdU Labeling and Detection Kit III (Sigma-Aldrich, St. Louis, MO, USA) following the manufacturer’s instructions. Results were expressed relative to the untreated control (UC), set as 100%.

### 2.14. The Muse^®^ Flow Cytometer Analysis

The cells were labeled using the Muse^®^ Annexin V & Dead Cell Assay Kit (Merck, Kenilworth, NJ, USA) according to the manufacturer’s instructions

### 2.15. Statistical Analysis

The results were expressed as the means ± standard deviation (SD). Statistical analyses were performed using Statistica v. 13.3 software (Tulsa, OK, USA). The data were subjected to an analysis of variance (ANOVA) followed by Duncan’s or Tukey’s post hoc test, which were performed to determine whether differences were significant at *p* ≤ 0.05. *t*-test was applied to compare effect of flaxseed extracts on the proliferation of cancer cells with *p* ≤ 0.05 was regarded as significant. Based on obtained results for the Muse^®^ flow cytometer analysis, one-way analysis of variance (ANOVA) was carried out. The Duncan post hoc test was used for testing the differences and *p* ≤ 0.05 was regarded as significant.

## 3. Results

### 3.1. Proximate Composition

The content of chemical compounds of the studied flaxseed per fresh weight (FW) significantly differed (*p* ≤ 0.05). The dry matter content of brown flaxseed (92.84 ± 0.78%) was statistically significantly (*p* ≤ 0.05) different from that of defatted brown flaxseed (87.99 ± 0.08%) and from that of defatted golden flaxseed (88.58 ± 0.03%) (Table 1).

Of the plant samples tested, defatted brown flaxseed and defatted golden flaxseed demonstrated the highest crude ash contents (5.44 ± 0.16 g/100 g FW and 5.38 ± 0.09 g/100 g FW, respectively), with the difference being statistically significant (*p* ≤ 0.05) (Table 1). A statistically significant difference (*p* ≤ 0.05) in ash content was determined between brown flaxseed (3.12 ± 0.11 g/100 g FW), defatted brown flaxseed (5.44 ± 0.16 g/100 g FW) and defatted golden flaxseed (5.38 ± 0.09 g/100 g FW).

The crude fat content of the brown flaxseed tested (40.48 ± 1.16 g/100 g FW) was the highest, differing significantly (*p* ≤ 0.05) from all other samples analyzed (Table 1). No significant differences (*p* > 0.05) were observed in the crude fat content between defatted brown flaxseed (15.12 ± 0.19 g/100 g FW) and defatted golden flaxseed (15.20 ± 0.86 g/100 g FW). The latter exhibited the lowest crude fat content of all the samples analyzed (Table 1).

Analysis of the fatty acid profile in brown and golden flaxseed showed the presence of seven different fatty acids (Table 2). Oleic acid (a monounsaturated fatty acid, MUFA), linoleic acid (a polyunsaturated fatty acid, PUFA) and particularly α-linolenic acid (a polyunsaturated fatty acid, PUFA) were dominant fatty acids in all analyzed samples of flaxseed. The most abundant fatty acid in all analyzed samples was α-linolenic acid (ALA). Its highest concentration was found in defatted golden flaxseed (48.96%), followed by golden flaxseed (43.97%) and brown flaxseed (43.85%), with the lowest concentration found in defatted brown flaxseed (38.26%). The highest content of oleic acid was observed in brown flaxseed (25.65%), while the lowest was in golden flaxseed (20.59%). Intermediate values of oleic acid were displayed by ground samples (23.40% in defatted golden flaxseed and 24.14% in defatted brown flaxseed). Linoleic acid, an omega-6 fatty acid, was present at the highest level in golden flaxseed (21.84%) and defatted brown flaxseed (20.30%), while brown and defatted golden flaxseed contained lower amounts (14.19% and 14.96%, respectively). Saturated fatty acids, such as palmitic acid (C16:0) and stearic acid (C18:0), constituted a smaller portions of the total fatty acids. The palmitic acid content ranged from 6.40% (golden) to 8.21% (defatted brown), while the stearic acid content varied from 4.31% (defatted golden) to 7.73% (brown). Other minor components included vaccenic acid, found in low amounts (0.77–1.13%), and γ-linolenic acid, which was only detected in defatted brown flaxseed (1.31%).

There were no statistically significant differences (*p* > 0.05) in total protein content in the analyzed flaxseed samples (Table 1). The analyzed plant samples were characterized by the fallowing total protein content: brown flaxseed 9.79 ± 0.47 g/100 g FW, defatted brown flaxseed 9.63 ± 0.16 g/100 g FW, golden flaxseed 9.78 ± 1.03 g/100 g FW, and defatted golden flaxseed 9.66 ± 0.27 g/100 g FW (Table 1).

Significant statistical differences (*p* ≤ 0.05) in the content of assailable carbohydrates were found between the tested plant materials (Table 1). The highest values were obtained for defatted brown flaxseed (46.41 ± 2.09 g/100 g FW) and defatted golden flaxseed (41.83 ± 1.47 g/100 g FW). These values were statistically significantly different (*p* ≤ 0.05) from each other and from those of the other samples. There were no significant differences (*p* ≤ 0.05) in digestible carbohydrates content between brown (24.42 ± 1.47 g/100 g FW) and golden (21.38 ± 1.47 g/100 g FW) flaxseed.

The analysis showed that the dietary fiber content in brown flaxseed (22.18 ± 2.07 g/100 g FW) and defatted brown flaxseed (23.53 ± 2.08 g/100 g FW) was not statistically significantly different (*p* ≤ 0.05) (Table 1). Statistically significant differences (*p* ≤ 0.05) were observed when the content of this nutrient was compared to that in brown and golden flax varieties (Table 1).

### 3.2. Mineral Content

The main minerals detected in the flaxseed samples were magnesium, calcium, sodium, and potassium, while the minor or trace elements included iron, zinc, copper, and manganese (Table 3). The calcium content of defatted brown flaxseed was statistically significantly (*p* ≤ 0.05) higher than that of the other samples (153.91 ± 3.54 mg/100 g FW). Magnesium content, as measured by atomic absorption spectrometry, was 521.7 ± 7.13 mg/100 g FW in defatted golden flaxseed and 457.43 ± 8.01 mg/100 g FW in defatted brown flaxseed. The highest statistically significant (*p* ≤ 0.05) potassium content was found in Defatted golden flaxseed exhibited the highest statistically significant (*p* ≤ 0.05) potassium content (1173.61 ± 8.59 mg/100 g FW), followed by defatted brown flaxseed (1116.14 ± 6.86 mg/100 g FW). The highest (*p* ≤ 0.05) iron content was found in defatted brown flaxseed at 23.02 ± 0.07 mg/100 g FW. The zinc content of defatted golden and brown flaxseed was higher than that of the non-defatted counterparts (Table 3). The manganese content of defatted brown flaxseed was 5.53 ± 0.15 mg/100 g FW.

### 3.3. Selected Bioactive Compounds Content

The concentration of vitamin C was similar across all analyzed samples, ranging from 4.29 ± 0.32 mg/100 g FW in defatted brown flaxseed to 5.75 ± 0.64 mg/100 g FW in golden flaxseed (Table 4). Brown flaxseed exhibited the highest content of total carotenoids (32.36 ± 4.43 mg/100 g FW), which was statistically significant (*p* ≤ 0.05) (Table 4). Defatted forms of flaxseed had a lower carotenoids content than non-defatted forms (Table 4).

Table 4 also shows the results of the analysis of the total polyphenols content of the tested plant materials. Defatted brown flaxseed exhibited the highest concentration of total polyphenols (1562.56 ± 29.83 mg ChlA eq./100 g FW, *p* ≤ 0.05), whereas non-defatted brown flaxseed showed the lowest content of these bioactive compounds (293.83 ± 19.12 mg ChlA eq./100 g FW, *p* ≤ 0.05) (Table 4). No statistically significant (*p* > 0.05) differences were found between golden flaxseed (398.33 ± 15.18 mg ChlA eq./100 g FW) and defatted golden flaxseed (435.30 ± 16.48 mg ChlA eq./100 g FW (Table 4).

Analysis of the polyphenol profile showed statistically significant differences (*p* ≤ 0.05) in the concentration of individual polyphenolic compounds in the examined plant materials (Table 5). The analyses identified 14 different polyphenolic compounds in golden flaxseed, 20 in defatted golden flaxseed, 17 in brown flaxseed and 22 in defatted brown flaxseed (Table 5). Defatted brown flaxseed exhibited the highest (*p* ≤ 0.05) concentration of polyphenolic compounds detected relative to the other types of flaxseed (183.78 mg/100 g FW), while golden flaxseed had the lowest (*p* ≤ 0.05) content of these compounds (22.10 mg/100 g FW) (Table 5).

Brown flaxseed showed higher concentrations of kaempferol (9.14 ± 0.13 mg/100 g FW), apigenin (8.51 ± 0.14 mg/100 g FW) and hispidulin (13.66 ± 0.27 mg/100 g FW) compared to the other types of flaxseed (Table 5). Among the phenolic acids, the highest amount of gallic acid (17.57 ± 0.0 mg/100 g FW, *p* ≤ 0.05) was determined in defatted brown flaxseed. Catechin (65.6 ± 0.12 mg/100 g FW) and epicatechin (11.61 ± 0.03 mg/100 g FW) appeared to be the flavonoids in the highest (*p* ≤ 0.05) concentrations in defatted brown flaxseed (Table 5). The highest (*p* ≤ 0.05) contents of carnosic acid were found in the brown and golden flaxseed samples, at 22.01 ± 1.31 mg/100 g FW and 11.4 11.4 ± 0.41 mg/100 g FW, respectively (Table 5). Defatted brown and defatted golden flaxseed showed higher carnosol content at 44.13 ± 0.26 mg/100 g FW and 29.21 ± 1.32 mg/100 g FW, respectively, compared to brown and golden flaxseeds (Table 5).

### 3.4. Antioxidant Capacity

Table 6 shows the results of the experiments investigating the antioxidant activity of the analyzed flaxseed samples. Defatted brown flaxseed exhibited the highest antioxidant activity (measured by three methods), with an ABTS^●^* radical scavenging activity of 829.17 ± 68.89 µmol Trolox/g FW, which was statistically significant (*p* ≤ 0.05) (Table 6). No significant statistical (*p* > 0.05) differences were found between the other types of plant material (Table 6). Analysis of the antioxidant activity of the plant material carried out using the FRAP reagent showed that the results obtained for brown (258.09 ± 0.94 µmol Trolox/g FW) and defatted brown flaxseed (1672.26 ± 25.32 µmol Trolox/g FW) were statistically significantly (*p* ≤ 0.05) different from the results obtained for golden (314.43 ± 2.90 µmol Trolox/g FW) and defatted golden flaxseed (322.59 ± 3.94 µmol Trolox/g FW) (Table 6).

### 3.5. Adhesion of Lactic Acid Bacteria

**Figure 1:**
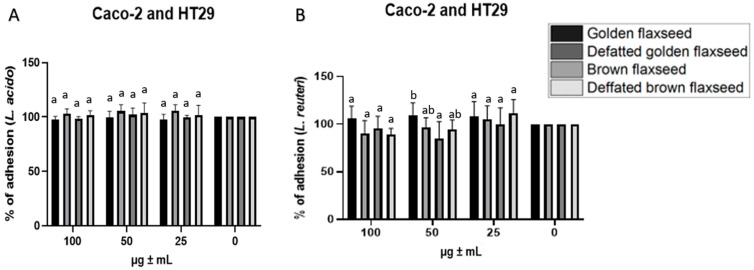
Adhesion of *Lactobacillus acidophilus* (**A**) and *Lactobacillus reuteri* (**B**) to a mixed co-culture of Caco-2/HT-29 cell lines in the presence of flaxseed extracts (25, 50 and 100 μg/mL). Values are expressed as a percentage of bacterial adhesion after a 2 h exposure to the extract concentrations compared to the control. Result are expressed as mean ± SD (*n* = 9) and were evaluated using a two-factor ANOVA with Tukey’s multiple comparisons test. a, b, statistically significant differences *p* ≤ 0.05 between samples within a single concentration.

**Figure 2:**
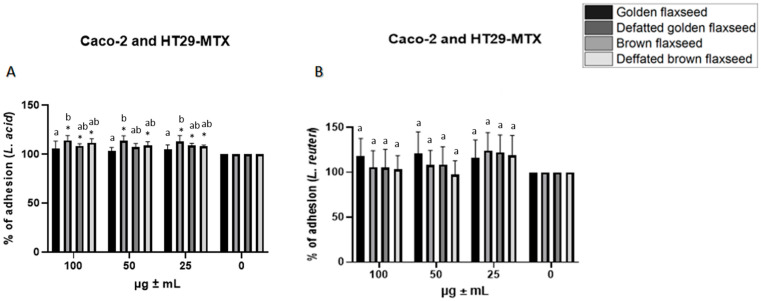
Adhesion of *Lactobacillus acidophilus* (**A**) and *Lactobacillus reuteri* (**B**) to a mixed co-culture of Caco-2/HT-29-MTX cell lines in the presence of flaxseed extracts (25, 50 and 100 μg/mL). Values are expressed as a percentage of bacterial adhesion after a 2 h exposure to the extract concentrations compared to the control. Result are expressed as mean ± SD (*n* = 9) and were evaluated using a two-factor ANOVA with Tukey’s multiple comparisons test. * statistically significant differences *p* ≤ 0.05 between samples and the untreated control. a, b, statistically significant differences *p* ≤ 0.05 between samples within a single concentration.

For the adhesion assays, flaxseed extracts at concentrations of 100, 50 and 25 µg/mL were introduced to the cell lines alongside with *Lactobacillus acidophilus* and *Lactobacillus reuteri* (Figure 1 and Figure 2). Adhesion was tested using two cellular models: Caco-2 and HT-29 and Caco-2, and HT-29-MTX (specifically cultured for mucin production). No statistically significant differences (*p* > 0.05) were observed between the strains, the samples, or the applied concentrations for the Caco-2 and HT-29 model with *Lactobacillus acidophilus* (Figure 1A). For the *Lactobacillus reuteri* strain, adhesion decreased by up to 10% as the concentration increased. A statistically significant difference (*p* ≤ 0.05) was observed in the ethanol extract of defatted brown flaxseed at concentrations between 100 and 25 µg/mL. Differences were also noted at a concentration of 50 µg/mL between golden flaxseed and brown flaxseed extracts (Figure 1B).

Further differences were observed in the Caco-2 and HT-29-MTX cell lines. Statistically significant differences (*p* ≤ 0.05) were found for *Lactobacillus acidophilus* between concentrations and extracts compared to the control (Figure 2A). No significant difference (*p* > 0.05) was recorded for *Lactobacillus reuteri* between concentrations and compared to the control (Figure 2B).

### 3.6. Anti-Inflammatory Response in Murine Macrophages on Their Produce Nitric Oxide (NO)

**Figure 3:**
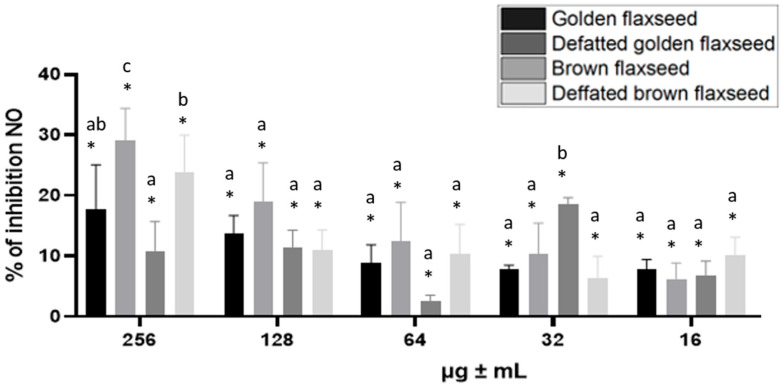
The ability of flaxseed extracts (16, 32, 64, 128 and 256 μg/mL) to reduce nitric oxide production in RAW264.7 mouse macrophages stimulated with LPS (1 μg/mL). The effect of the flaxseed extracts was measured after 24 h of cell incubation. Result are expressed as mean ± SD (*n* = 9), standardized to the treated control (with LPS) set as 100% and were evaluated using a two-factor ANOVA with Tukey’s multiple comparisons test. * statistically significant differences *p* ≤ 0.05 between samples and treated control. a–c, statistically significant differences *p* ≤ 0.05 between samples within a single concentration.

The subsequent effect under investigation was the impact of flaxseed extracts on the inhibition of nitric oxide production in RAW 264.7 mouse macrophages when stimulated with LPS. Figure 3 shows that the ability to inhibit nitric oxide production decreases with lower concentrations, depending on the sample. An exception was brown flaxseed extract at a concentration of 32 µg/mL, which exhibited higher inhibitory ability than at higher concentrations (Figure 3). Golden and brown flaxseed extracts, at a concentration of 256 µg/mL, decreased nitric oxide production in RAW 264.7 cells more effectively than their defatted counterparts.

### 3.7. Impact on Cancer Cell Proliferation and Apoptosis

**Figure 4:**
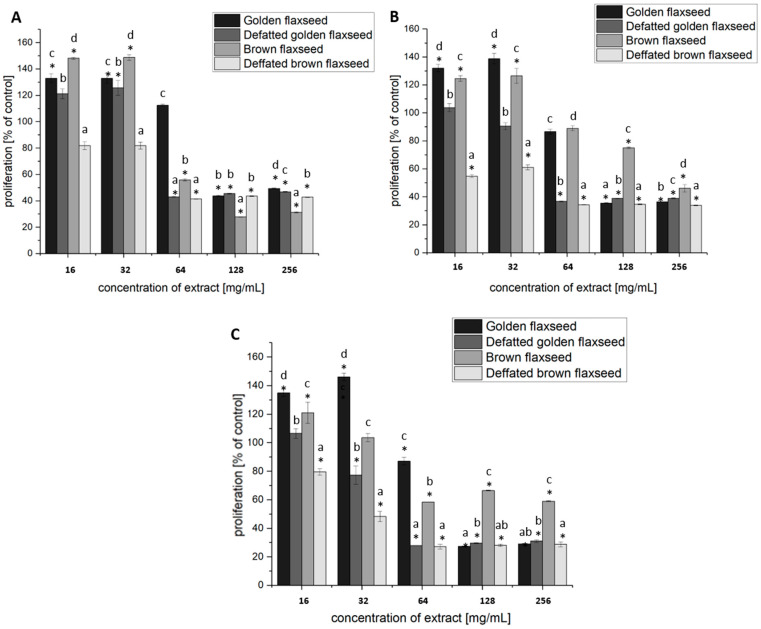
Effect of flaxseed extracts (16, 32, 64, 128 and 256 μg/mL) on the proliferation of MCF-7 breast cancer cells in a Cell Proliferation ELISA, BrdU test. The effect of the flaxseed extracts was measured after 24 h (**A**), 48 h (**B**) and 72 h (**C**) of cell incubation. Results are expressed as means ± SD (*n* = 9), standardized to the untreated control set as 100%. * statistically significant differences (based on a Student *t*-test) *p* ≤ 0.05 between samples and the untreated control. a–d, statistically significant differences (based on ANOVA and Duncan’s a posteriori test) *p* ≤ 0.05 between samples within a single concentration.

BrDU labeling results indicated that flaxseed extracts inhibited the proliferation of the studied breast cancer cell line in a dose- and time-dependent manner (Figure 4). Incubation of MCF-7 breast cancer cells for 24, 48, and 72 h with flaxseed extracts at concentrations of 64, 128, and 256 μg/mL significantly affected cell proliferation (*p* ≤ 0.05, Figure 4). Proliferation was reduced by approximately 72% after 24 h (brown flaxseed), 66% after 48 h (defatted brown flaxseed) and 72% after 72 h (golden and defatted brown flaxseed) (*p* ≤ 0.05, Figure 4). The most prominent results were obtained with defatted brown flaxseed, which effectively reduced the proliferation of MCF-7 breast cancer cells at almost every concentration used. The exception was the 24 h incubation of breast cancer cells at concentrations of 16 or 32 μg/mL, for which no significant (*p* > 0.05) differences were demonstrated compared to the untreated control (Figure 4A). More effective reduction in the proliferation of MCF-7 breast cancer cell line was observed after 24 h of incubation with the defatted brown flaxseed extract, compared to the brown flaxseed extract. This effect was maintained during 48 and 72 h incubation of cells with the tested extracts (Figure 4).

Treatment of MCF-7 breast cancer cells with flaxseed extracts significantly reduced cell viability and increased the populations of apoptotic cells, compared to the untreated control (Table 7, Figure S1). In the untreated control, the majority of cells remained viable (84.81% ± 1.59) with a low apoptotic fraction (14.18% ± 1.48). As expected, staurosporine (STS) induced the strongest apoptotic response, leaving only 8.63% ± 3.20 of the cells viable and yielding 91.20% ± 3.21 apoptotic cells, predominantly in the early stage (60.97% ± 5.85). Golden flaxseed and defatted golden flaxseed significantly (*p* ≤ 0.05) decreased viability (38.68% ± 2.13 and 37.87% ± 1.13, respectively) and increased total apoptosis (46.52% ± 1.94 and 43.60% ± 2.02), with a predominance of late apoptosis. Brown flaxseed extract exerted less pronounced effect (32.78% ± 0.78 apoptotic cells), while defatted brown flaxseed demonstrated enhanced potency (52.97% ± 4.19 apoptotic cells), similar to golden flaxseed extracts.

## 4. Discussion

Flaxseed (*Linum usitatissimum* L.) is characterized by its high proximate composition, with oil content typically ranging between 35% and 65%. The seeds are rich in polyunsaturated fatty acids, particularly α-linolenic acid (omega-3) and linoleic acid (omega-6), as well as other fatty acids such as oleic, stearic, and palmitic acids. In addition to mentioned lipids, flaxseeds are a valuable source of protein, dietary fiber, and lignans, all of which contribute to their high nutritional value. The seeds also contain essential micronutrients, vitamins, and phenolic compounds with recognized antioxidant properties [17]. The diverse chemical composition of flaxseed underscores its potential as a functional food with numerous health benefits. Although its general nutritional profile is well documented, compositional variations exist among different flaxseed varieties and depending on processing methods

The dry matter content of the brown flaxseed in this study differed significantly (*p* ≤ 0.05) from that of defatted brown flaxseed and defatted golden flaxseed (Table 1). According to Sargi et al. [18], the dry matter content of brown and golden flaxseed was 93.48% and 93.27%, respectively. Comparable results were obtained by Jain et al. [19], who determined a dry matter content of 95.83% in flaxseed samples.

The results indicated that defatted plant materials exhibited the highest crude ash content (Table 1). Comparable values were reported by Jain et al. [19], who analyzed two types of flaxseed and reported ash content values ranging from 2.91 to 3.00 g/100 g FW. Similarly, Sargi et al. [18] found that the ash content of brown and golden flaxseed was 2.63 g/100 g FW, 2.84 g/100 g FW, respectively.

In terms of crude fat, Jain et al. [19], reported an average of 26.09 g/100 g FW, while Sargi et al. [18], determined total fat contents of 38.13 g/100 g FW for brown flaxseed and 37.57 g/100 g FW for golden flaxseed. The results of the present study confirm that α-linolenic acid is the predominant fatty acid in flaxseed. A comparison of the fatty acid profiles of brown and golden flaxseed varieties reveals a similarity in the composition of fatty acid profiles, with ALA accounting for between 48.4% and 58.9% of the total fatty acids present [4]. In accordance with the findings of the present study, Yang et al. [20] reported oleic acid as the second most abundant fatty acid, followed by linoleic acid. Teneva et al. [21] determined comparable results, with oleic acid accounting for ~30% and linoleic acid for 10–20% of total fatty acids. The lower concentration of ALA in the defatted brown flaxseed (38.26%) relative to the defatted golden flaxseed (48.96%) suggests that the process of defatting may result in differential alterations to the fatty acid profile, contingent on the specific variety of flaxseed. Similarly, intermediate oleic acid values in defatted samples may be indicative of partial degradation or redistribution of fatty acids during processing, as also suggest by Oeffner et al. [22].

Slightly different results for protein content in flaxseed were obtained by Sargi et al. [18] and Jain et al. [19], who determined protein levels of 17.26 g/100 g FW and 24.42 g/100 g FW the brown flaxseed and 23.24 g/100 g FW for golden flaxseed. Flaxseed is naturally gluten-free, making it a potential protein source for individuals with gluten intolerance or celiac disease. The digestibility of flaxseed proteins depends on their structural matrix, i.e., whether they are present alone or bound with other nutrients such as mucilages or oils. Studies have demonstrated that flaxseed proteins are highly bioavailable, with an estimated digestibility of approximately 89% [23,24], highlighting their potential as a valuable protein source in human nutrition. Flaxseed is not considered a major carbohydrate source, as its total carbohydrate content is approximately 29%, with simple sugars and starch accounting for around 1%. However, it contains two important mucilaginous polysaccharides—rhamnogalacturonan and arabinoxylan—that contribute to its functional properties [23]. Rhamnogalacturonan, an acidic polysaccharide belonging to the pectin group, constitutes about 25% of total flaxseed polysaccharides. It is composed of L-fructose, L-rhamnose, D-galacturonic acid and D-galactose. Rhamnogalacturonan enhances the rheological properties of gels, improves emulsion stability, and exhibits anti-inflammatory, anticancer and immunomodulatory effects. Arabinoxylan, another major polysaccharide, is found in most seeds and is composed of arabinose, galactose, and xylose. In vivo studies have confirmed its ability to increase the activity of immune cells, including macrophages, monocytes and natural killer (NK) cells [24]. Both rhamnogalacturonan and arabinoxylan have been shown to coat the mucous membranes of the esophagus, stomach and intestines [25]. This protective effect is thought to result from their capacity to buffer against the damaging effects of gastric acid, thereby maintaining appropriate pH levels along the gastrointestinal tract. Owing to these properties, flaxseed has been used in the treatment of gastric and duodenal ulcers, as well as for alleviating constipation, due in part to its anti-inflammatory and mucilage-rich composition [24,26,27]. Significant differences in digestible carbohydrate content were observed between the analyzed plant materials, with defatted brown and defatted golden flaxseed showed the highest values, whereas no significant differences (*p* > 0.05) were found between brown and golden flaxseed (Table 1). Similar results were obtained by Mancharkar et al. [28], who determined the carbohydrate content to be 26.1 g/100 g FW. Sargi et al. [18], found comparable values, reporting 28.29 g/100 g FW and 29.61 g/100 g FW for brown and golden flaxseed, respectively.

The soluble fiber content of flaxseed has been reported to range from approximately 4.3% to 8.6%, while the insoluble fraction accounts for approximately 12.8–17.1%. The soluble fraction primarily consists of mucilage, whereas the insoluble fraction contains mainly lignin and cellulose [29,30]. The present findings are in line with previous findings by Noreen et al. [31], who determined total fiber content of 25.8 g/100 g FW, and by Jain et al. [19], who obtained a value of 16.75 g/100 g FW.

Flaxseed is considered a good plant-based source of essential nutrients, including macroelements and microelements. The major minerals identified in the analyzed studied samples were magnesium, calcium, sodium and potassium. Furthermore, the analysis revealed the presence of minor minerals or trace elements, including iron, copper, and manganese in the samples (Table 3). The exact amount of calcium in flaxseed can vary slightly based on factors like the specific type of flaxseed, whether it is whole or ground, and how it is processed. The obtained results showed that the content of calcium in defatted brown flaxseed was higher than in other samples (Table 3). Similar values were obtained by Jain et al. [19], Mancharkar et al. [28], and Sharma and Saini [32] who stated that flaxseeds contained an average calcium content of 200–230 mg/100 g FW. The results showed that defatted golden and brown flaxseed were the richest source of magnesium compared to the non-fatty counterparts (Table 3). Similar results were obtained by Noreen et al. [31] and Sharma and Saini [32]. An analysis of the mineral composition showed that the level of potassium was the highest compared to the other described minerals, in all analyzed samples. The content of this mineral in defatted golden as well as brown flaxseed was found to be the highest and was found to be higher than in results obtained by Noreen et al. [31] and Sharma and Saini [32]. Iron is a micro-nutrient of significance in flaxseeds, which contributes to their nutritional value. While flaxseeds are not among the highest sources of iron, they still play a role in providing this essential mineral. The defatted brown flaxseed exhibited the highest levels of iron among the other samples analyzed (Table 3). The present study has similar results as Sharma and Saini [32], who found this mineral content in flaxseed 22  ±  0.32 mg/100 g. Different values were obtained by Jain et al. [19], who reported that flaxseeds contained an average of 4.66 mg iron/100 g FW. Different conclusions were also reached by Mancharkar et al. [28], determining 2.83 mg iron/100 g FW. The results obtained demonstrated that the zinc content of the defatted golden and brown flaxseed was higher than that of the non-fatty counterparts (Table 3). Other authors who examined flaxseed reported varying values [31,32]. The findings of the present study demonstrated that the manganese content of defatted brown flaxseed was 5.53 ± 0.15 mg/100 g FW, which is comparable to the results reported by Noreen et al. [31] and Sharma and Saini [32]. These findings underscore the nutritional value of flaxseed, particularly as a significant source of essential minerals. The variations in mineral content reported in different studies suggest that factors such as soil composition, growing conditions, and processing methods play a crucial role in determining the final nutrient profile of flaxseed.

Flaxseed contains notable amounts of lipid-soluble micronutrients, particularly tocopherols and carotenoids. The tocopherol content in flaxseed oil ranges from 460 to 610 mg/kg, with γ-tocopherol being the predominant homolog, thereby substantially enhancing its antioxidant activity [33]. Additionally, carotenoids, which are responsible for the red, orange, and yellow colors in many seeds and fruits, are present in flaxseed at levels ranging from 0.7 to 3.1 mg/kg. However, some studies have reported β-carotene levels reaching up to 77 mg/kg in flaxseed oil [34]. These compounds play a crucial role in preventing photo-oxidation, a process of particular importance given flaxseed’s high unsaturated lipid content. The vitamins and carotenoids present in flaxseed have been demonstrated to contribute to its health-promoting properties and antioxidant capabilities [35].

The findings of the present study demonstrate that the tested varieties and forms of flaxseeds differed significantly in terms of the content of selected bioactive compounds. As demonstrated in Table 4, the vitamin C content in the analyzed samples was similar. Differences in content were observed for carotenoids and phenolic compounds. Compared to full-fat samples, defatted flaxseed exhibited a lower carotenoid content but a higher concentration of polyphenols. As demonstrated in the study by Emam et al. [36], a higher concentration of vitamin C was obtained in flaxseed cultivated in Egypt than in the present research. Katare et al. [29] reported that flaxseed contain 0.5 mg/100 g vitamin C. However, flaxseed is not a significant source of this vitamin. Instead, it is a good source of niacin and vitamin E, primarily in the form of tocopherol, which possesses strong antioxidant properties. The tocopherol content in flaxseed has been shown to range from 39.5 to 50 mg per 100 g [37]. Sufficient vitamin E intake has been linked to a lower risk of cardiovascular diseases, Alzheimer’s disease, and certain types of cancer [30]. It has been demonstrated that when flaxseed is subjected to defatting, a substantial proportion of its lipid content is eliminated. This process has been observed to result in a decline in carotenoid levels. This phenomenon has been observed in other oil-rich seeds and plant materials where the fat fraction contains a high concentration of lipophilic compounds, including carotenoids [38]. However, the extent of the reduction would depend on the processing method used for defatting and whether some carotenoids remain bound to proteins or other components of the seed [39]. The flaxseed samples analyzed in this study exhibited significant concentrations of total carotenoids, which is in alignment with the observations reported by Mueed et al. [40]. These researchers documented that the carotenoid content of flaxseed ranges from 0.7 to 3.1 mg/kg. A study analyzing 40 flaxseed varieties found total carotenoid contents ranging from 45.2 to 310.84 µg/100 g dry weight (DW), with lutein being the predominant carotenoid, constituting approximately 70% to 80% of the total carotenoid content [41]. The higher total carotenoid content observed in the analyzed samples of brown flaxseed in comparison to golden flaxseed may be indicative of differences in seed coat biochemistry and antioxidant capacity. Brown flaxseed shows higher levels of several compounds, including protein, fiber, tocopherols, and phenolics, in comparison to golden flaxseed. The observed differences are attributed to the genetic and biochemical properties of the seed coat, which in turn influence the synthesis and subsequent accumulation of these compounds [42,43]. It has been demonstrated that carotenoids have the capacity to protect developing seeds from photooxidative and oxidative stress. The findings of Wu et al. [41] suggests that carotenoid accumulation is strongly genotype-dependent and may be associated with oxidative stress tolerance during the process of seed maturation.

The analysis of total polyphenols revealed differences among the studied flaxseed samples (Table 4). In particular, defatted brown flaxseed exhibited the highest concentration of the compounds under investigation, whereas whole brown seeds contained the lowest levels. This observation aligns with the findings reported by Teh et al. [44] and Yadav et al. [45], who documented total polyphenol contents of 406.67 mg/100 g FW and 354.82 mg/100 g FW, respectively. The results of the polyphenol profile analysis by HPLC were consistent with those obtained in the total polyphenol assay using the Follin-Ciocalteu reagent. The fraction of flavonoids and diterpenes accounted for the majority of the labeled polyphenolic compounds in all case. As demonstrated in Table 5, flavonones and diterpenes were predominant in brown flaxseed, whereas diterpenes were dominant in golden and defatted golden flaxseed, and flavonoids prevailed in defatted brown flaxseed. The lowest concentrations of phenolic acids were determined in all of the plant samples analyzed (Table 5). Huang et al. [46] validated the antioxidant activity of flaxseed polyphenols. Their study examined the content of phenolic acids and flavonoids in flaxseeds, highlighting notable differences in the types and amounts of polyphenols across various flaxseed varieties. The most prevalent phenolic compounds identified were sinapic acid, ferulic acid, kaempferol, and quercitrin. Among the phenolic acids, caffeic acid, ferulic acid and *p*-coumaric are listed as the most abundant in flaxseed. Katare et al. [29] reported that the content of phenolic acid in flaxseed is approximately 35–70 mg/100 g. Among the phenolic acids, caffeic acid, ferulic acid, and *p*-coumaric were the most abundant in flaxseed. The quantity of total flavonoids in flaxseed varies depending on the cultivation conditions and variety, with concentrations ranging from 30 to 71 mg/100 g [47,48]. The range of phenolic acids found in Canadian flaxseed varieties varies between 790–1030 mg per 100 g, with notable constituents including chlorogenic acid, *p*-hydroxy benzoic acid, ferulic acid, vanillic acid, and coumaric acid [49].

Wang et al. [50], isolated two novel compounds from the diterpene group (L27 and L28) from the seeds of the plant *Euphorbia lathyris*, a member of the wolfberry family (*Euphorbiaceae Juss*.), which is related to the flax family (*Linaceae*). The authors performed a cytotoxicity assay against breast and liver cancer cell lines, which showed a strong ability of the isolated compounds to reduce the viability of cancer cells. Loussouarn et al. [51] demonstrated that carnosic acid and carnosol possess antioxidant and anticancer properties. In a study by Mira-Sanchez et al. [52] demonstrated the content of these compounds in individual extracts prepared from rosemary at concentrations of 21.31 ± 0.11%; 20.75 ± 0.07%; 35.75 ± 0.15% and 80.81 ± 0.35%. The results obtained by the researchers are similar to the values presented in Table 5, which gives reason to believe that flaxseed may have similar properties.

The present investigation employed three assays to evaluate the antioxidant activity of plant samples: ABTS^●^*, DPPH, and FRAP reagents. Higher antiradical activity was observed with by ABTS^●^* compared to the DPPH and FRAP, which Kusznierewicz et al. [53] also described. The antioxidant activity of the flaxseed samples varied depending on type and processing method. The results obtained from both ABTS^●^* and DPPH assays showed that defatted brown flaxseed exhibited the highest radical scavenging activity, which was several-fold higher than that observed in the other samples. A similar trend was observed for the FRAP assay, where defatted brown flaxseed showed the strongest reducing power, while brown flaxseed exhibited the weakest response. These results highlights the potential of defatted brown flaxseed as a promising source of antioxidants. Sargi et al. [15] reported different values of antioxidant activity of flaxseed (3.38 ± 0.09 mmol Trolox/g FW), while Kučka et al. [54] observed a wider range (0.04–4.43 mmol Trolox/g FW), suggesting that factors such as growing conditions, processing methods, and genetic variations may significantly influence the antioxidant properties of flaxseed. The findings of this study are in alignment with the results of Deme et al. [55], in which the antioxidant values of flaxseed samples ranged from 152.5 to 305.9 µmol Trolox/g FW.

The adhesion of *Lactobacillus acidophilus* was found to be enhanced following incubation with defatted golden flaxseed, in comparison to golden flaxseed (Figure 4). Previous studies have indicated that the lipid content of substrates can influence the adhesion properties of probiotics. While omega-3 fatty acids, such as those found in flaxseed oil, are known to possess anti-inflammatory properties and support gut health, their effect on probiotic adhesion can be mixed. Some studies suggest that the lipophilic nature of these fats may prevent optimal interaction between the probiotic bacteria and the substrate. For instance, a study by Kretzschmar and Manefield [56] found that the lipid content of plant extracts could hinder bacterial attachment to surfaces. Possibly due to the hydrophobic character of lipids interfering with hydrophilic interactions between probiotics and plant fibers or mucilage. Lipids can mask or modify the surface properties of the substrate, thereby reducing the available surface area for bacterial attachment [57]. On the other hand, alpha-linolenic acid, a major component of flaxseed oil, has been demonstrated to promote the adhesion of probiotics. Research indicated that treatment with ALA increased the adhesive activity of *Lactobacillus acidophilus* and *Bifidobacterium bifidum* to colonic epithelial cells in a dose-dependent manner [58]. In addition, ALA promoted the adhesion of probiotics into colonic epithelial cells in an in vitro model, indicating a beneficial effect towards the intestinal microbiota. Dybka-Stępień et al. [59] found that the polysaccharides in flaxseed mucilage were highly effective in promoting the adhesion of probiotic strains to the intestinal epithelium, suggesting that mucilage might serve as a prebiotic for beneficial bacteria. It is hypothesized that defatted flaxseed, with its reduced lipid content, may allow the mucilage to have a more direct and enhanced effect on bacterial adhesion. Similarly, Sungatullina et al. [60] demonstrated that the incorporation of flaxseed mucilage into the growth medium of various *Lactobacillus* strains increased their adhesion to intestinal epithelial cells.

Improved probiotic survival and adhesion can lead to better colonization in the gut, supporting gastrointestinal health, immune modulation, and enhanced production of beneficial metabolites such as short-chain fatty acids and polyphenols [61]. Furthermore, research has demonstrated that flaxseed mucilage and oil cake extracts have the capacity to augment antioxidant activity and stimulate the synthesis of bioactive compounds, including lipase and α-glucosidase inhibitors. This, in turn, serves to further enhance the functional and health-promoting value of probiotic foods [60,62]. The combined application of flaxseed extracts and probiotic strains has been shown to exert a favorable influence on the composition of gut microbiota, to enhance immune responses, and to promote enhanced metabolic health outcomes in both animal and in vitro studies [63,64].

The present study demonstrated that golden and brown flaxseed extracts exhibited a significant inhibitory effect on nitric oxide (NO) production in LPS-stimulated RAW 264.7 cells (Figure 3). Flaxseed extract demonstrates significant in vitro nitric oxide (NO) inhibition through multiple molecular mechanisms, with protein hydrolysate fractions showing the most direct inhibitory effect. Mechanistically, flaxseed protein hydrolysate fractions can reduce endothelial nitric oxide synthase (eNOS) activity through mixed-type inhibition [65]. This inhibition occurs by altering the structure of calmodulin and consequently reducing enzyme activity. Additional studies have confirmed the NO-inhibitory potential of flaxseed extracts. As demonstrated by Chera et al. [66], a dose-dependent reduction in NO levels was observed in models of macrophages. This finding suggests that bioactive compounds present in flaxseed may interfere with inflammatory signaling pathways. This effect is likely to be mediated through the modulation of transcription factors such as NF-κB (nuclear factor kappa-light-chain-enhancer of activated B cells), which plays a central role in regulating inducible nitric oxide synthase (iNOS) expression and NO production [67,68]. Consistently, α-linolenic acid has been shown to exert anti-inflammatory effects by downregulating iNOS expression and inhibiting NO production in activated macrophages [69]. Flaxseed peptides have been demonstrated to possess antioxidant properties by neutralizing hydroxyl radicals (OH·) and reducing nitric oxide (NO) production in macrophages [70]. Similarly, a polysaccharide fraction extracted from flaxseed hull, consisting of multiple monosaccharides, has been shown to exert strong antioxidant and anti-inflammatory properties, likely mediated via the NO and mitogen-activated protein kinase (MAPK) pathways [71].

The antioxidant and anticancer effects of flaxseed are attributable to the synergistic action of bioactive compounds, which neutralize free radicals, reduce oxidative stress, and modulate the signaling pathways involved in cancer progression [46,72].The results obtained from this study indicate that flaxseed extracts inhibited the proliferation of MCF-7 breast cancer cells in a dose- and time-dependent manner, significantly reducing cell viability and inducing apoptosis (Figure 4, Table 7). It is noteworthy that defatted brown flaxseed extract demonstrated the most pronounced antiproliferative and pro-apoptotic effects. Research has demonstrated the anticancer properties of compounds present in flaxseed, showing effectiveness against various cancer types, including breast, cervical, ovarian, colon, leukemia, and melanoma cells [73]. Studies also indicate that linseed derivatives, such as linseed oil, can inhibit melanoma metastasis and slow the growth of lung cancer, supporting their protective role against cancer. Interestingly, linseed oil treatment did not affect the growth of non-cancerous cells, suggesting that linseed and its products selectively target malignant cells without causing harm to normal cells [74]. In vitro studies show that flaxseed extract causes dose-dependent decreases in MCF-7 cell viability by triggering reactive oxygen species production, mitochondrial membrane potential loss, and caspase cascade activation [75]. Similarly, flaxseed sprouts induce apoptosis in both estrogen-receptor-positive (MCF-7) and estrogen-receptor-negative (MDA-MB-231) breast cancer cells while upregulating p53 mRNA expression, without affecting normal mammary epithelial cells [76]. Flaxseed oil specifically inhibits cancer cell growth and induces apoptosis through mitochondrial dysfunction and DNA fragmentation in various cancer cell lines, including MCF-7 cells [73]. Clinical evidence supports above findings, as a randomized controlled trial demonstrated that dietary flaxseed consumption in postmenopausal breast cancer patients significantly increased tumor apoptosis by 30.7% while reducing cell proliferation markers [77]. Flaxseed bioactives promote apoptosis in cancer cells through both intrinsic and extrinsic pathways. In the mitochondrial (intrinsic) pathway, orbitides and lignans increase the Bax/Bcl-2 ratio, induce cytochrome c release, and activate caspases-9 and -3, leading to DNA fragmentation and cell death in gastric, leukemia, and breast cancer cell lines [6,73,78,79,80]. The action of linusorb is closely linked to the induction of apoptosis in cancer cell lines, including through the activation of the extrinsic pathway (death receptor pathway), increasing the expression of Fas, death receptor 5 (DR5) and tumor necrosis factor receptor 1 (TNF-R1) protein in cancer cells [73]. Flaxseed compounds also modulate pro- and anti-apoptotic gene expression, upregulating *BAK1*, *CASP10*, *Fas*, and *TNF*, while downregulating *BCL2*, potentially via MAPK/p38 and protein kinase C delta (PKCδ) signaling pathways [81].

At the molecular level, the observed effects can be partly attributed to major polyphenolic compounds identified in flaxseed extracts—catechin, epicatechin, and carnosol—which exert significant bioactivity through complementary mechanisms, contributing to both nitric oxide (NO) inhibition in RAW264.7 macrophages and apoptosis induction in MCF-7 breast cancer cells. Catechin has been demonstrated to inhibit nitric oxide (NO) production in lipopolysaccharide-stimulated RAW264.7 macrophages. This suppression is achieved by decreasing inducible nitric oxide synthase (iNOS) expression and activity through the inhibition of NF-κB and p38 MAPK pathways, consequently leading to a reduction in inflammation and oxidative stress [82]. Furthermore, it has been observed that catechin compounds interact with NADPH oxidase subunits, thereby further contributing to their inhibitory effects on NO [83]. In MCF-7 cells, epicatechin has been shown to induce apoptosis primarily via mitochondrial-mediated pathways, involving increased reactive oxygen species (ROS) generation, loss of mitochondrial membrane potential, activation of caspase-independent apoptotic cascades, and DNA fragmentation. This pro-apoptotic effect is accompanied by the upregulation of pro-apoptotic proteins, such as Bad and Bax [84]. Carnosol, a naturally occurring diterpene derived from rosemary, has been shown to inhibit NO production in macrophages by suppressing NF-κB signaling, leading to a reduction in iNOS expression and a consequent decrease in inflammation [85]. In breast cancer cells, carnosol has been shown to promote apoptosis through several mechanisms, including the ROS-dependent induction of endoplasmic reticulum stress (ER), p38 MAPK activation, and increased expression of markers of the unfolded protein response [86]. In addition, carnosol has been demonstrated to regulate the expression of proteins associated with apoptosis by means of upregulating the catalytic subunit of glutamate-cysteine ligase (GCLC) and cyclooxygenase-2 (COX-2), whilst concomitantly downregulating the anti-apoptotic protein Bcl-2. This, in turn, results in mitochondrial dysfunction and the subsequent activation of intrinsic apoptotic pathways [87]. Taken together, these findings suggest that the polyphenolic composition of flaxseed extracts can modulate inflammatory signaling in macrophages and induce programmed cell death in breast cancer cells. The synchronized actions of major polyphenols provide a mechanistic rationale for the observed inhibition of NO and the induction of apoptosis, thus highlighting their potential anti-inflammatory and anticancer effects.

## 5. Conclusions

Flaxseed is a nutrient-rich plant source known for its high content of essential minerals, bioactive compounds, and potential health benefits. This study analyzed the proximate and mineral composition, polyphenols and carotenoids content, antioxidant capacity, probiotic adhesion properties, anti-inflammatory and anticancer effects of various flaxseed samples, including golden and brown seed and their defatted forms. The results demonstrated that defatted brown flaxseed had the highest concentrations of calcium, magnesium, potassium, iron, and zinc, contributing to its superior nutritional profile. Polyphenols content analysis revealed significant variations between flaxseed types, with defatted brown flaxseed exhibiting the highest total polyphenol content and the greatest abundance of flavonoids and diterpenes. Antioxidant activity, measured using multiple assays, was significantly higher in defatted brown flaxseed compared to other samples. Additionally, defatted flaxseed extracts enhanced probiotic adhesion. In anti-inflammatory assays, flaxseed extracts demonstrated the ability to reduce nitric oxide production in macrophages, suggesting potential applications in managing inflammation-related conditions. This study highlights the significant antiproliferative effects of flaxseed extracts on breast cancer cells, demonstrating a dose- and time-dependent inhibition of MCF-7 cell proliferation. Among the tested samples, defatted brown flaxseed extract showed the most pronounced anticancer activity, effectively reducing cancer cell growth at nearly all concentrations and inducing the highest levels of apoptosis. The observed effects can be attributed to the high levels of polyphenols, carotenoids, and other bioactive compounds, which exhibit strong antioxidant and tumor-suppressing properties. Flaxseed, a substantial source of biologically active compounds, may serve as a promising product for the development of functional foods and nutraceuticals aimed at supporting antioxidant, anti-inflammatory, and anticancer functions. The in vitro findings reported in this study provide a rationale for future in vivo investigations aimed at confirming the physiological efficacy and safety of flaxseed-derived bioactives.

## Figures and Tables

**Table 1:** Basic chemical composition of golden and brown flaxseed (*Linum usitatissimum*).

Type of Flaxseed	Dry Matter (g)	Ash (g/100 g FW)	Crude Fat (g/100 g FW)	Protein (g/100 g FW)	Dietary Fiber (g/100 g FW)	Digestible Carbohydrates (g/100 g FW)
Golden	91.88 ^b^ ± 0.12	3.9 ^a^ ± 0.07	36.34 ^b^ ± 0.45	9.78 ^a^ ± 1.03	28.09 ^b^ ± 2.45	21.38 ^a^ ± 1.47
Defatted golden	88.58 ^a^ ± 0.03	5.38 ^b^ ± 0.09	9.66 ^a^ ± 0.27	9.66 ^a^ ± 0.27	27.93 ^b^ ± 1.75	41.83 ^b^ ± 1.47
Brown	92.84 ^b^ ± 0.78	3.12 ^a^ ± 0.11	40.48 ^c^ ± 1.16	9.79 ^a^ ± 0.47	22.18 ^a^ ± 2.07	24.42 ^a^ ± 1.47
Defatted brown	87.99 ^a^ ± 0.08	5.44 ^b^ ± 0.16	15.12 ^a^ ± 0.19	9.63 ^a^ ± 0.16	23.53 ^a^ ± 2.08	46.41 ^b^ ± 2.09

Result are expressed as mean ± SD (*n* = 3). Mean values with different letters (a–c) within the each column are statistically different *p* ≤ 0.05 (based on ANOVA and Duncan’s a posteriori test). FW, fresh weight.

**Table 2:** Fatty acid profile of brown and golden flaxseed (*Linum usitatissimum*).

Type of Flaxseed	Palmitic Acid C16:0	Stearic Acid C18:0	Oleic Acid C18:1	Vaccenic Acid C18:1	Linoleic Acid C18:2	α-Linolenic Acid C18:2	γ-Linolenic Acid C18:2
Golden	6.4 ^a^ ± 0.01	6.24 ^b^ ± 0.03	20.59 ^a^ ± 0.06	0.95 ^a,b^ ± 0.03	21.84 ^d^ ± 0.05	43.97 ^b^ ± 0.07	0.0 ^a^ ± 0.0
Defatted golden	7.61 ^b^ ± 0.08	4.31 ^a^ ± 0.01	23.4 ^b^ ± 0.15	0.77 ^a^ ± 0.2	14.96 ^b^ ± 0.05	48.96 ^c^ ± 0.2	0.0 ^a^ ± 0.0
Brown	7.73 ^b^ ± 0,1	7.73 ^c^ ± 0.06	25.65 ^d^ ± 0.23	0.86 ^a,b^ ± 0.27	14.19 ^a^ ± 0.05	43.85 ^b^ ± 0.29	0.0 ^a^ ± 0.0
Defatted brown	8.21 ^c^ ± 0.03	6.64 ^b^ ± 0.05	24.14 ^c^ ± 0.01	1.13 ^b^ ± 0.01	20.30 ^c^ ± 0.04	38.26 ^a^ ± 0.08	1.31 ^b^ ± 0.04

Result are expressed as mean ± SD (*n* = 3). Mean values with different letters (a–d) within the each column are statistically different *p* ≤ 0.05 (based on ANOVA and Duncan’s a posteriori test). FW, fresh weight.

**Table 3:** Mineral composition of golden and brown flaxseed (mg/100 g FW).

Type of Flaxseed	Calcium	Magnesium	Potassium	Sodium	Iron	Zinc	Manganese	Copper
Golden	98.07 ^b^ ± 0.42	386.14 ^c^ ± 1.3	853.84 ^c^ ± 3.56	29.5 ^b^ ± 8.47	19.77 ^c^ ± 0.37	8.17 ^c^ ± 0.39	3.94 ^b^ ± 0.03	1.38 ^d^ ± 0.01
Defatted golden	94.96 ^b^ ± 0.84	521.7 ^a^ ± 7.13	1173.61 ^a^ ± 8.59	28.39 ^b^ ± 2.82	21.67 ^b^ ± 1.13	9.43 ^a^ ± 0.18	3.87 ^b^ ± 0.12	1.8 ^b^ ± 0.01
Brown	97.63 ^b^ ± 2.02	313.01 ^d^ ± 0.85	683.46 ^d^ ± 8.38	15.16 ^c^ ± 0.25	19.62 ^c^ ± 0.55	5.41 ^d^ ± 0.03	2.85 ^c^ ± 0.21	1.49 ^c^ ± 0.01
Defatted brown	153.91 ^a^ ± 3.54	457.43 ^b^ ± 8.01	1116.14 ^b^ ± 6.86	43.04 ^a^ ± 2.26	23.02 ^a^ ± 0.07	8.83 ^b^ ± 0.06	5.53 ^a^ ± 0.15	2.15 ^a^ ± 0.02

Result are expressed as mean ± SD (*n* = 3). Mean values with different letters (a–d) within the each column are statistically different *p* ≤ 0.05 (based on ANOVA and Duncan’s a posteriori test). FW, fresh weight.

**Table 4:** Selected bioactive compounds contents in golden and brown flaxseed.

Type of Flaxseed	Vitamin C (mg/100 g FW)	Total Carotenoids (mg/100 g FW)	Total Polyphenols (mg ChlA eq./100 g FW)
Golden	5.75 ^b^ ± 0.64	15.57 ^b^ ± 1.39	398.33 ^b^ ± 15.18
Defatted golden	5.65 ^a,b^ ± 0.63	13.98 ^b^ ± 0.56	435.3 ^b^ ± 16.48
Brown	4.34 ^a,b^ ± 1.08	32.36 ^c^ ± 4.43	293.83 ^c^ ± 19.12
Defatted brown	4.29 ^a^ ± 0.32	9.91 ^a^ ± 0.59	1562.56 ^a^ ± 29.83

Result are expressed as mean ± SD (*n* = 3). Mean values with different letters (a–c) within the each column are statistically different *p* ≤ 0.05 (based on ANOVA and Duncan’s a posteriori test). FW, fresh weight.

**Table 5:** Concentration of polyphenolic compounds in brown and golden flaxseed (mg/100 g FW).

Polyphenolic Compounds	Type of Flaxseed			
	Golden	Defatted Golden	Brown	Defatted Brown
Gallic acid	ND	ND	ND	17.57 ^a^ ± 0.0 H
4-Hydroxybenzoic acid	0.5 ^b^ ± 0.0 A	0.96 ^c^ ± 0.0 AB	0.4 ^a^ ± 0.0 A	1.55 ^d^ ± 0.0 CD
Vanillic acid	0.24 ^a^ ± 0.0 A	0.38 ^b^ ± 0.0 A	0.24 ^a^ ± 0.0 A	0.58 ^c^ ± 0.0 AB
Syringic acid	ND	ND	0.28 ^a^ ± 0.0 A	0.82 ^b^ ± 0.0 AB
Chlorogenic acid	0.49 ^a^ ± 0.0 A	1.56 ^d^ ± 0.0 AB	0.58 ^b^ ± 0.07 A	0.84 ^c^ ± 0.0 AB
Caffeic acid	ND	0.26 ^a^ ± 0.0 A	0.25 ^a^ ± 0.0 A	0.47 ^b^ ± 0.0 A
*p*-Coumaric acid	0.35 ^b^ ± 0.0 A	0.23 ^a^ ± 0.0 A	0.25 ^a^ ± 0.0 A	0.52 ^c^ ± 0.01 A
Ferulic acid	0.49 ^a^ ± 0.0 A	1.27 ^d^ ± 0.0 AB	0.58 ^b^ ± 0.0 A	1.15 ^c^ ± 0.0 BC
Sinapinic acid	0.26 ^a^ ± 0.0 A	0.31 ^c^ ± 0.0 A	ND	0.29 ^b^ ± 0.0 A
Rosmarinic acid	ND	0.37 ^a^ ± 0.0 A	ND	0.85 ^b^ ± 0.0 AB
Catechin	ND	5.24 ^a^ ± 0.46 C	ND	65.6 ^b^ ± 0.12 J
Epicatechin	2.01 ^a^ ± 0.0 B	2.89 ^a^ ± 0.0 B	2.13 ^a^ ± 0.01 A	11.61 ^b^ ± 0.03 G
Naringin	ND	0.78 ^a^ ± 0.0 AB	0.91 ^a^ ± 0.0 A	1.49 ^b^ ± 0.05 CD
Rutin	ND	0.54 ^a^ ± 0.0 AB	0.65 ^b^ ± 0.01 A	0.76 ^c^ ± 0.0 AB
Kaempferol	1.0 ^a^ ± 0.0 A	0.98 ^a^ ± 0.0 AB	9.14 ^b^ ± 0.13 B	1.95 ^a^ ± 0.23 D
Myricetin	ND	0.83 ^a^ ± 0.0 AB	ND	1.61 ^b^ ± 0.0 CD
Hesperidin	0.48 ^a^ ± 0.0 A	0.66 ^b^ ± 0.0 AB	0.48 ^a^ ± 0.0 A	1.19 ^c^ ± 0.01 BC
Apigenin	0.28 ^a^ ± 0.0 A	0.25 ^a^ ± 0.0 A	8.51 ^b^ ± 0.14 B	1.11 ^a^ ± 0.01 F
Hispidulin	2.11 ^b^ ± 0.0 B	1.01 ^a^ ± 0.0 AB	13.66 ^d^ ± 0.27 C	3.55 ^c^ ± 0.09 E
Acacetin	0.52 ^b^ ± 0.0 A	0.7 ^d^ ± 0.0 AB	0.47 ^a^ ± 0.0 A	0.67 ^c^ ± 0.0 AB
Carnosol	0.47 ^a^ ± 0.02 A	29.21 ^c^ ± 1.32 E	14.7 ^b^ ± 0.61 C	44.13 ^d^ ± 0.26 I
Carnosic acid	11.4 ^a^ ± 0.41 C	14.6 ^a^ ± 0.05 D	22.01 ^b^ ± 1.31 D	21.31 ^b^ ± 0.02 I

Result are expressed as mean ± SD (*n* = 3). Statistically significant differences (*p* ≤ 0.05) in the concentration of polyphenolic compounds between the various types of flaxseed within the each row are indicated by letter code a–d. Mean values with different letters (A–J) within the each column are statistically different (*p* ≤ 0.05), indicating differences between individual polyphenols within one type of flaxseed (based on ANOVA and Duncan’s a posteriori test). FW, fresh weight.

**Table 6:** Antioxidant activity of brown and golden flaxseed measured by ABTS^●^*, DPPH and FRAP assays (µmol Trolox/g FW).

Type of Flaxseed	ABTS^●^*	DPPH	FRAP
Golden	83.47 ^a^ ± 8.11	57.89 ^a^ ± 1.37	314.43 ^b^ ± 2.90
Defatted golden	88.45 ^a^ ± 1.85	61.24 ^a^ ± 3.77	322.59 ^b^ ± 3.94
Brown	73.64 ^a^ ± 4.65	53.31 ^a^ ± 8.78	258.09 ^a^ ± 0.94
Defatted brown	414.59 ^b^ ± 34.45	368.09 ^b^ ± 88.83	1672.26 ^c^ ± 25.32

Result are expressed as mean ± SD (*n* = 3). Mean values with different letters (a–c) within the each column are statistically different *p* ≤ 0.05 (based on ANOVA and Duncan’s a posteriori test). FW, fresh weight.

**Table 7:** Cell apoptosis results in MCF-7 breast cancer cells treated with flaxseed extracts. Apoptosis profiling and apoptotic cell counts were obtained using flow cytometry (Muse^®^ Cell Analyzer) and the Muse^®^ Annexin V and Dead Cell Assay Kit.

	Live	Early Apoptotic	Late Apoptotic	Total Apoptotic
UC	84.81 ^d^ ± 1.59	3.10 ^a^ ± 0.32	11.07 ^a^ ± 1.41	14.18 ^a^ ± 1.48
STS	8.63 ^a^ ± 3.20	60.97 ^d^± 5.85	30.23 ^c^ ± 3.38	91.20 ^e^ ± 3.21
Golden flaxseed	38.68 ^b^ ± 2.13	8.07 ^b,c^ ± 0.21	38.45 ^d^ ± 2.11	46.52 ^c,d^ ± 1.94
Defatted golden flaxseed	37.87 ^b^ ± 1.13	6.23 ^a^ ± 0.45	37.37 ^d^ ± 2.03	43.60 ^c^ ± 2.02
Brown flaxseed	55.35 ^c^ ± 0.87	11.30 ^c^ ± 0.57	21.48 ^b^ ± 0.55	32.78 ^b^ ± 0.78
Defatted brown flaxseed	36.13 ^b^ ± 1.03	4.39 ^a,b^ ± 1.49	48.58 ^e^ ± 2.82	52.97 ^d^ ± 4.19

UC, untreated control; STS, staurosporine. The final concentration of the extracts was 128 μg/mL. The effect of the flaxseed extract was measured after 48 h of incubation of cells. Results are expressed as means ± SD (n = 3). Mean values with different letters (a–e) within the each column are statistically different *p* ≤ 0.05 (based on ANOVA and Duncan’s a posteriori test).

## Data Availability

The original contributions presented in this study are included in the article/Supplementary Materials. Further inquiries can be directed to the corresponding author.

## Supplementary Materials

The following supporting information can be downloaded at: https://www.mdpi.com/article/10.3390/nu17213407/s1, Materials and Methods: Fatty acid profile, Minerals content, HPLC analysis of polyphenols, Figure S1. Cell apoptosis results of MCF-7 cancer cells at treated with experimental flaxseed extracts. The apoptosis profiling and apoptotic cell counts were obtained using flow cytometry (Muse^®^ Cell Analyzer) and Muse^®^ Annexin V and Dead Cell Assay Kit.
