# Supplementary material for: Compositional and Functional Analysis of Golden and Brown Flaxseed: Nutrients, Bioactive Phytochemicals, Antioxidant Activity, and Cellular Responses
Source: Nutrients. 2025 Oct 29;17(21):3407. doi: 10.3390/nu17213407 (PMC12608315; doi:10.3390/nu17213407)
Supplement: Supplementary file 1 [file nutrients-17-03407-s001.zip › nutrients-3917772-supplementary.pdf]

Material and Methods

Fatty acid profile

Free fatty acids were converted to methyl esters using boron trifluoride in methanol (BF_3/MeOH).The fatty acid methyl esters were isolated with hexane and analyzed on a Shimadzu GC-17A-QP5050 system (Shimadzu, Japan) equipped with a Supelco SP-2560 capillary column (30 m × 0.25 mm × 0.25 μm; Bellefonte, PA, USA). Helium was used as the carrier gas at a flow rate of 1.8 mL/min. The injector temperature was set to 245 °C, and 1 μL of sample was injected. The column temperature program started at 60 °C (5 min hold), increased by 5 °C/min to 220 °C, and was maintained for 20 min.

Minerals content

Wet mineralization was performed in sealed vessels using a Mars Express microwave system with 5 mL of 65% HNO_3 at 170 °C for 15 min. The digests were diluted with deionized water prior to analysis. Metal ion concentrations were measured using flame Atomic Absorption Spectrometry (Varian AA240FS) with a Sample Introduction Pump System (SIPS-20). Gas flow rates were set at 14 L/min for air and 3.5 L/min for acetylene. Absorbance was recorded at the following wavelengths: Ca^{2+} – 422.7 nm, Mg^{2+} – 202.6 nm, K^{+} – 404.4 nm, Na^{+} – 589.6 nm, Fe^{3+} – 248.3 nm, Zn^{2+} – 213.9 nm, Cu^{2+} – 324.7 nm, Mn^{2+} – 279.5 nm.

HPLC analysis of polyphenols

The profile of polyphenolic compounds was determined by liquid chromatography (HPLC), in pre-prepared methanolic extracts, which were filtered through a 0.22 μm syringe filter into 2 ml vials. Analysis was performed using a Prominence-i LC-2030C 3D Plus system29 instrument (Shimadzu, Kyoto, Japan), equipped with a diode array detector (DAD). The separation of the mixture was carried out using a Luna Omega 5 μm Polar C18 column, 100 A, 250 x 10 mm column (Phenomenex, CA, USA) at 40 °C. The mobile phase consisted of 0.1% formic acid in water (A) and 0.1% formic acid in methanol (B), with a flow rate of 1.2 mL/min and an injection volume of 20 μL. The HPLC analysis was carried out under the following gradient program: solution B increased from 20% to 40% over 10 min, held at 40% for 10 min, increased from 40% to 50% in 10 min, 50→60% in 5 min, held at 60% for 5 min, increased to 70% in 5 min, 70→90% in 5 min, held at 90% for 5 min, then returned to 20% in 1 min and maintained for 4 min. The total analysis time was 60 min. Quantification of individual polyphenolic compounds was performed using standard curves, and analysis data were processed using Lab Solutions software (Shimadzu, Kyoto, Japan).

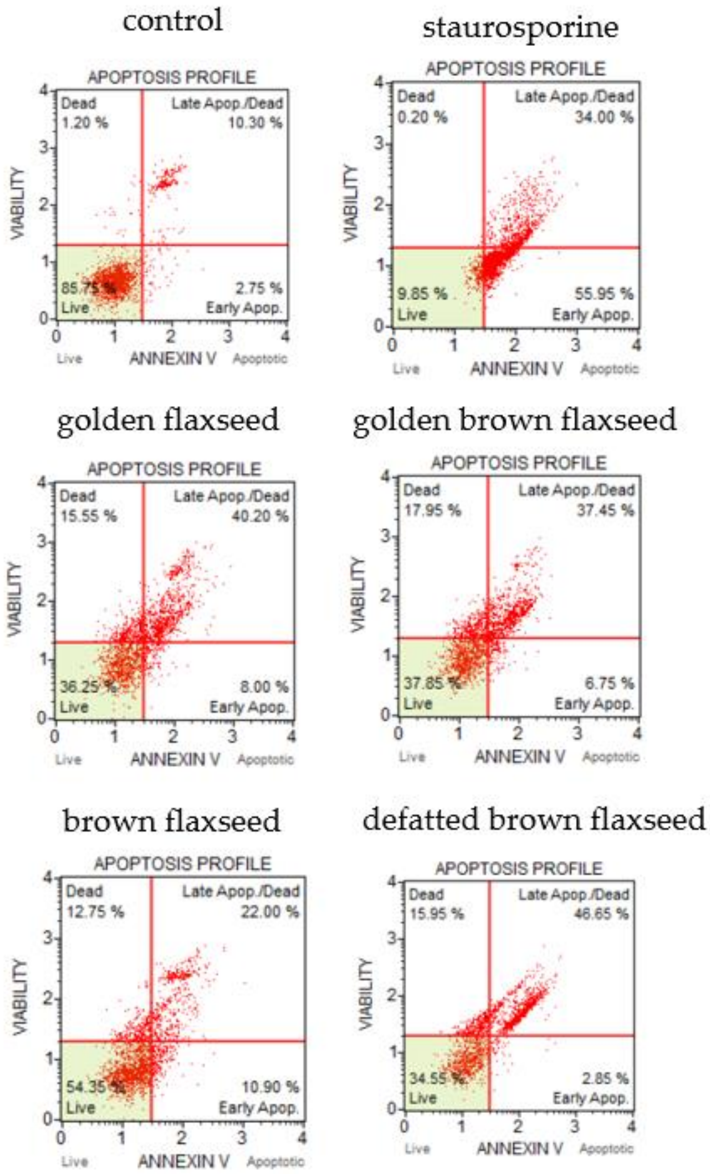

Figure S1. Cell apoptosis results of MCF-7 cancer cells at treated with experimental flaxseed extracts. The apoptosis profiling and apoptotic cell counts were obtained using flow cytometry (Muse® Cell Analyzer) and Muse® Annexin V and Dead Cell Assay Kit.
